# Shear Stress Inhibits Apoptosis of Ischemic Brain Microvascular Endothelial Cells

**DOI:** 10.3390/ijms14011412

**Published:** 2013-01-11

**Authors:** Shan Tian, Yulong Bai, Lin Yang, Xinggang Wang, Yi Wu, Jie Jia, Yulian Zhu, Yong Cheng, Pengyue Zhang, Junfa Wu, Nianhong Wang, Guang Xia, Hua Liao, Yuling Zhang, Xiafeng Shen, Huixian Yu, Yongshan Hu

**Affiliations:** 1 Department of Rehabilitation, Huashan Hospital, Fudan University, Shanghai 200040, China; E-Mails: shantian2008@hotmail.com (S.T.); baiyl@sohu.com (Y.B.); wuyi4000@163.com (Y.W.); shannonjj@126.com (J.J.); zyljully@yahoo.com (Y.Z.); zpy19802000@yahoo.com.cn (P.Z.); junfawu2002@yahoo.com.cn (J.W.); wnh2005@126.com (N.W.); zhangyuling1982@hotmail.com (Y.Z.); shenquan1@yahoo.com.cn (X.S.); huixianyu@126.com (H.Y.); 2 State Key Laboratory of Medical Neurobiology, Fudan University, Shanghai 200032, China; 3 Research Center, EYE & ENT Hospital of Fudan University, Shanghai 200031, China; E-Mail: yanglincd@126.com; 4 Shanghai Institute of Cardiovascular Diseases, Zhongshan Hospital, Fudan University, Shanghai 200032, China; E-Mails: wangxingning@outlook.com (X.W.); xiaguangyisheng@163.com (G.X.); liaohua226@163.com (H.L.); 5 Department of Cardiology, Zhengzhou Center Hospital, Zhengzhou University, Zhengzhou 450000, China; E-Mails: drchengyong@sina.com

**Keywords:** laminar shear stress, ischemic stroke, apoptosis, brain microvascular endothelial cells

## Abstract

As a therapeutic strategy for ischemic stroke, to restore or increase cerebral blood flow (CBF) is the most fundamental option. Laminar shear stress (LS), as an important force generated by CBF, mainly acts on brain microvascular endothelial cells (BMECs). In order to study whether LS was a protective factor in stroke, we investigated LS-intervented ischemic apoptosis of rat BMECs (rBMECs) through PE Annexin V/7-AAD, JC-1 and Hoechst 33258 staining to observe the membranous, mitochondrial and nuclear dysfunction. Real-time PCR and western blot were also used to test the gene and protein expressions of Tie-2, Bcl-2 and Akt, which were respectively related to maintain membranous, mitochondrial and nuclear norm. The results showed that LS could be a helpful stimulus for ischemic rBMECs survival. Simultaneously, membranous, mitochondrial and nuclear regulation played an important role in this process.

## 1. Introduction

Ischemic stroke is a common cause of death and long-term disability. Thrombolytic therapy and following rehabilitative exercise therapy represent efficient treatments and associate with restoring or increasing cerebral blood flow (CBF) [1–4]. Thrombolysis is necessary to eradicate the thrombosis blockage [2]. However, capillary autoregulation could not be recovered along with thrombolysis immediately [1,5], then exercise therapy could increase blood perfusion in penumbra through vascular blood output and redistribution [3,4,6–8] to alleviate remaining attenuated CBF. Animal studies showed that physical training improved endothelium-dependent vasodilation and CBF [7,8]. Clinical stroke survivors with aerobic exercise training had improved middle cerebral artery blood flow velocity and vasomotor reactivity [3].

Increasing CBF of the ischemic regions upregulates laminar shear stress (LS) on brain microvascular endothelial cells (BMECs) [1]. As the tangential component of the blood mechanical forces generated by CBF on endothelial cells [9],whether LS is a protective factors in stroke is uncertain, but its mechanotransduction has been extensively studied [9–13] and pointed out that endothelial cells could directly sense the alterations of stress to regulate vessel function and affect disease progression [10–12]. The most representative research of atherosclerosis [9,10,12] have demonstrated that LS potently regulates endothelial morphology, function, death and growth [10,12]. In particular, increased steady LS induced endothelial cell cycle arrest to inhibit apoptosis [9,13]. Consequently, we assumed that there was also a close relationship between LS and microvascular endothelial apoptosis. If endothelial apoptosis was alleviated, the damage of blood-brain barrier and entire brain tissue would be further reduced [14,15].

Meanwhile, signal transduction is the unique feature of mechanotransduction [10–12,16]. Extrinsic membrane-bound receptors of tumor necrosis factor (TNF), nitric oxide synthase (NOS) and MicroRNAs [8–10,12,16–18] could be activated by mechanical forces to lead cytoskeleton, cytoplasm, mitochondria, nuclei and many other organelles changes [10–12]. Furthermore, cellular apoptotic response is definitely characteristic with nuclear chromatin condensation, mitochondrial depolarization and membrane permeability [12,19]. Chromatin condensation is to be recognized as the most typical and reliable performance in general, while mitochondrial depolarization and membrane permeability are features of early apoptosis [10–12]. Therefore, the mature technologies of Hoechst33258, JC-1 and PE Annexin V/7-AAD staining were adopted to observe the three corresponding organelles’ dysfunction in apoptotic process [20–22]. Simultaneously, we detected protein and gene expression of some important factors related to membranous, mitochondrial and nuclear function. Tie-2 is one of two receptor tyrosine kinases expressed primarily in the vascular endothelium membrane [23,24] to participate in the regulation of capillary-like tubule formation and endothelial cells survival [23,25]. PI3K/Akt pathway is a classic antiapoptotic cascade to block nuclear condensation and DNA fragmentation by preventing a series of enzymatic reactions of the caspases family [12,26]. Bcl-2 resides in the outer mitochondrial wall to protect the integrity of mitochondria through regulating the release of cytochrome C, confronting the imbalance of energy metabolism, keeping permeability and preventing the collapse of mitochondrial membrane potentials [19]. Whether Tie-2, Akt and Bcl-2 participated in LS intervented-antiapoptosis needed certification.

Therefore, in order to verify if LS could affect the apoptosis of ischemic BMECs, we offered rat BMECs (rBMECs) LS by parallel-plate flow chamber [27]. This chamber was a device widely reported and used on the LS study *in vitro* [28,29]. Apoptosis and organelle injuries were detected by PE Annexin V/7-AAD, JC-1 and Hoechst33258 staining [20–22]. The expressions of Tie-2, Akt and Bcl-2 were detected by real-time PCR and Western blot. We suggested that appropriately increased LS inhibited apoptosis of ischemic rBMECs. Tie-2, Akt and Bcl-2, at least in part, attenuated a series of apoptotic events of membranous, mitochondrial and nuclear dysfunctions.

## 2. Results and Discussion

### 2.1. Characteristics of rBMECs

**Figure 1:**
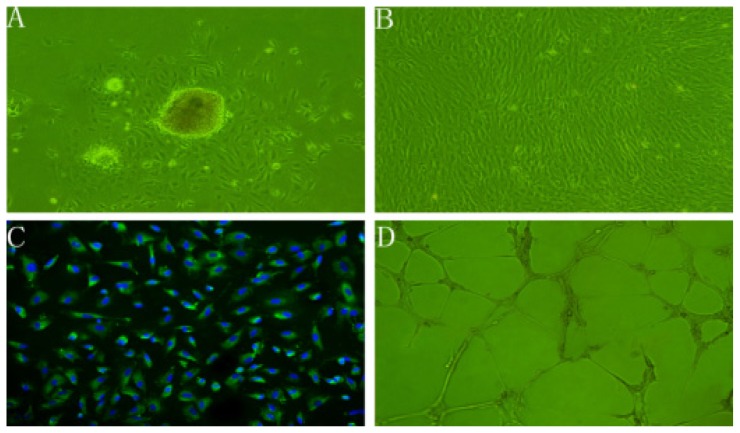
Characterization of rBMECs under microscope. (**A**) rBMECs at P0 were migrating out of the clusters of microvascular fragments (100×); (**B**) Confluent subcultured cells formed cobble-stone morphology (100×); (**C**) FITC-conjugated von Willebrand factor (VWF) and DAPI-stained nucleus excited out of green and blue fluorescence, correspondingly (200×); (**D**) Cells seeded onto Matrigel showed capillary-like tube formation (50×).

rBMECs in the initial passage predominantly grew out from clusters of microvascular fragments radially with swirling patterns and whorls (Figure 1A), but dispersed subcultured cells showed an uniform spindle-shaped morphology and assumed a cobble-stone like structure when confluenced (Figure 1B) [30,31]. Their strongly positive immunostaining for VWF was widely considered the most reliable endothelial cell-specific expressed protein marker (Figure 1C) [30]. In response to Matrigel matrix, rBMECs were stimulated to migrate as the process *in vivo* to form characteristic capillary tube-like structures (Figure 1D) [16,32]. On a gross level of morphology, biochemistry and function studies, the cells obtained were rBMECs.

### 2.2. rBMECs under Ischemic Condition

**Figure 2:**
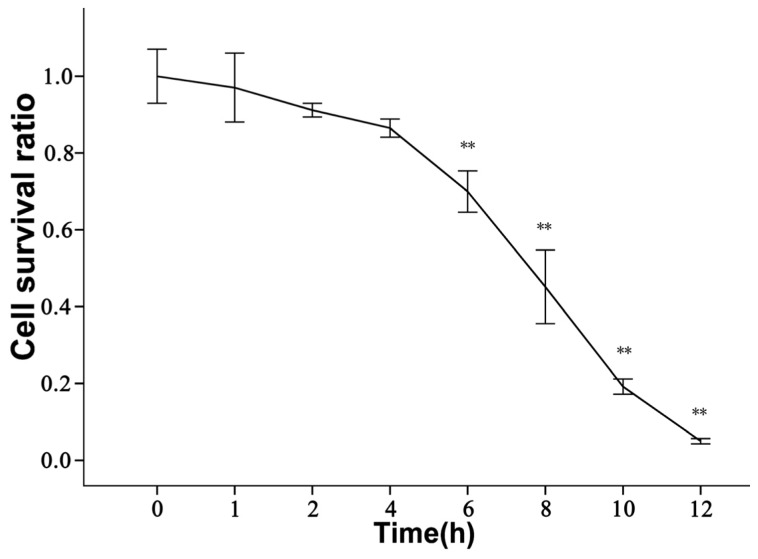
The ischemic rBMECs activity by WST-8 assay at ischemia 1 h, 2 h, 4 h, 6 h, 8 h, 10 h, 12 h was 97% ± 9%, 91% ± 2%, 86% ± 2%, 70% ± 5%, 45% ± 10%, 19% ± 2%, 5% ± 1%, respectively (** *p* < 0.01 *vs.* normal control group 0 h).

The activity of ischemic rBMECs was evaluated by WST-8 assay [33]. As shown in Figure 2, cell viability almost had no changes in 1–2 h (*p* > 0.05), began to decrease at 4 h, but had statistical difference until 6 h (*p* < 0.01). After 10 h, the majority of cells were apoptotic floating. rBMECs turned out to be susceptible to ischemia with time and 4–6 h was suitable for following LS intervention.

### 2.3. The LS Effects on Ischemic Cells with PE Annexin V/7-AAD, JC-1 and Hoechst33258 Staining

**Figure 3:**
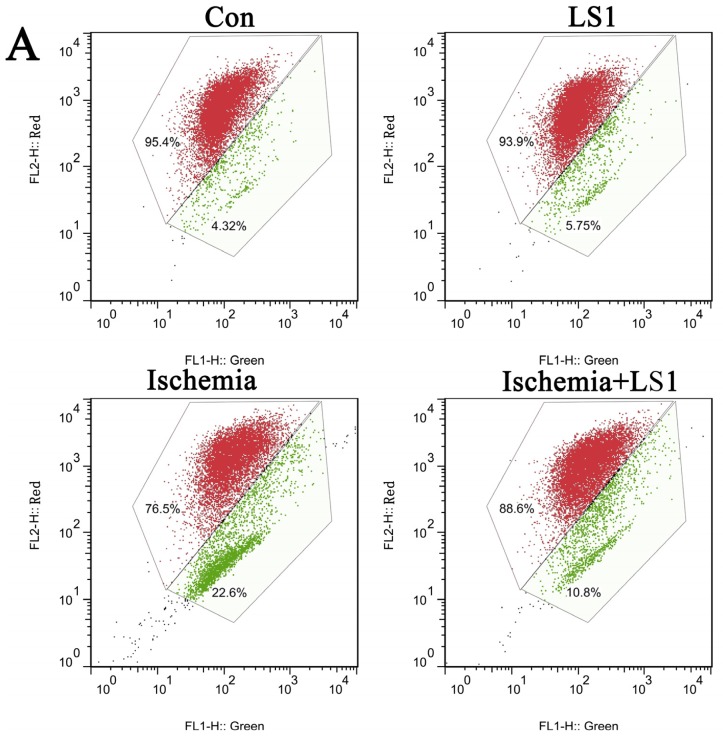

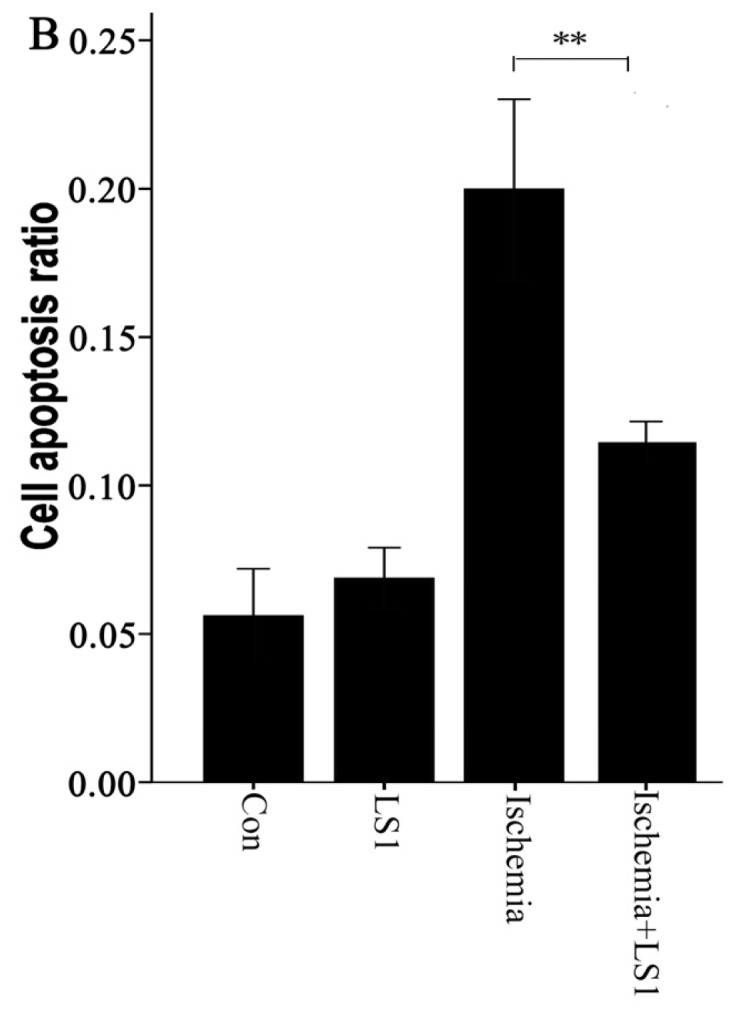
Effect of LS on mitochondrial membrane potentials and the apoptotic statistics between groups. (**A**) Mitochondrial membrane potentials were determined with JC-1 stinaing by flow cytometry (green: apoptosis; red: norm); (**B**) Compared with Con group, the apoptotic rate of LS1 group was not increased (*p* > 0.05); Compared with Ischemia group, the proportion of apoptotic cells in Ischemia+LS1 group were significantly decreased (** *p* < 0.01).

**Figure 4:**
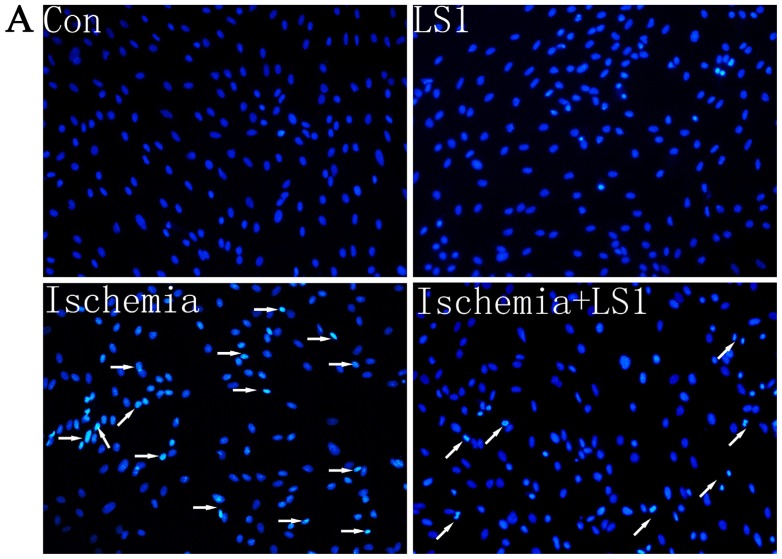

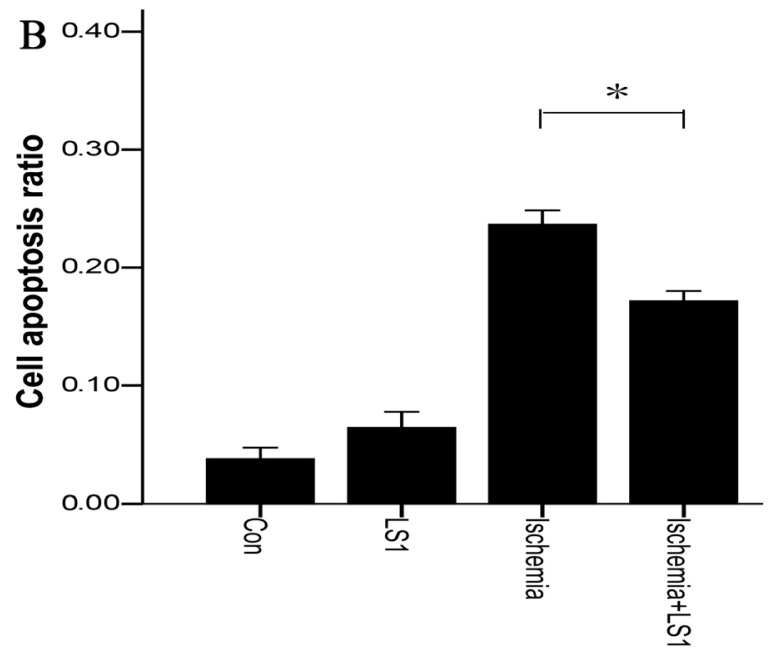
Effect of LS with Hoechst 33258 nuclear staining (200×) and the apoptotic statistics between groups. (**A**) adherent cells were incubated with Hoechst 33258 and phoned (Hoechst33258-positively bright blue indicated by arrows: apoptosis; dim blue: norm); (**B**) The apoptotic trend between groups was similar with JC-1 staining in Figure 3B (* *p* < 0.05).

**Figure 5:**
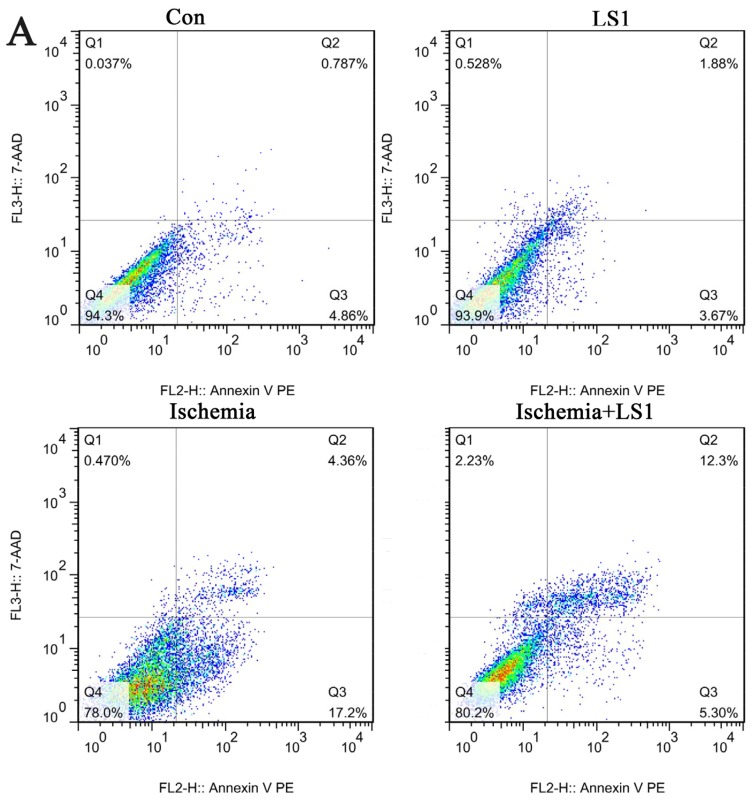

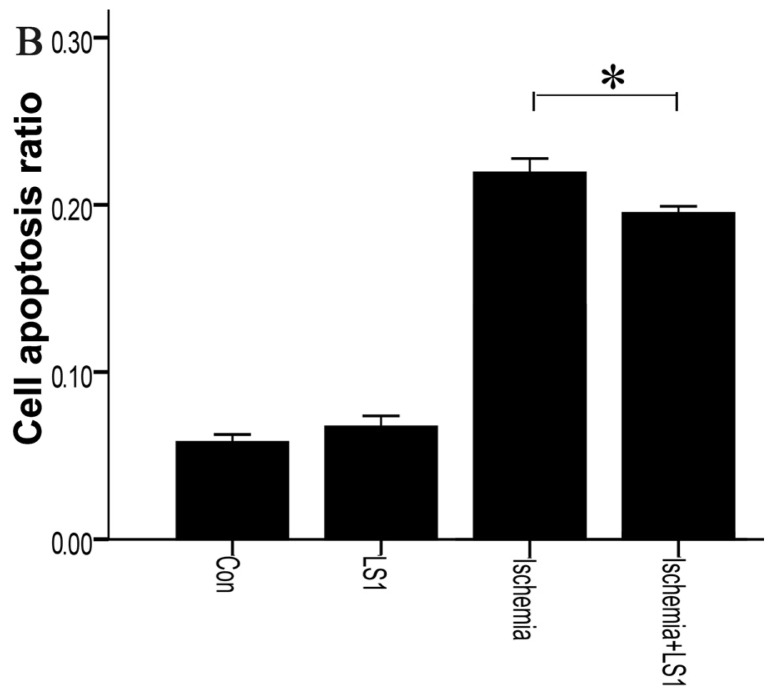
Effect of LS with membranous PE Annexin V/7-AAD staining and the apoptotic statistics between groups. (**A**) Intervented cells were incubated with PE Annexin V/7-AAD and analyzed by flow cytometry (Q2 + Q3: apoptosis; Q4: norm); (**B**) The apoptotic trend between groups was similar with JC-1 staining in Figure 3B (* *p* < 0.05).

rBMECs were divided into four groups: cells with static or 1 ± 0.05 dynes/cm^2^ LS in normal culture condition for 2 h (Con group or LS1 group); cells for ischemia 6 h (Ischemia group) and cells with extra 1 ± 0.05 dynes/cm^2^ LS for the last 2 h of ischemia group (Ischemia + LS1 group). Apoptosis was measured by JC-1, Hoechst33258 and PE Annexin V/7-AAD staining. These methods were not only the most commonly used indicators for apoptotic rates but also represented membranous, mitochondrial and nuclear dysfunctions respectively. Depolymerization of JC-1 aggregate in red fluorescence into JC-1 monomers in green fluorescence demonstrated mitochondrial depolarization [20]. The nucleus of apoptotic cells with chromatin condensation stained bright blue by Hoechst33258 whereas normal cells stained dim [21]. Positive PE Annexin V represented early apoptosis as well as positive 7-AAD was for late apoptosis [22]. In this study, the apoptotic analysis through three methods were parallel: the apoptotic proportion changed little if giving normal cells LS for 2 h (*p* > 0.05), whereas under ischemic condition, extra stress would obviously increase the survival rate (* *p* < 0.05 or ** *p* < 0.01) (Figures 3, 4 and 5). These results indicated that the given LS with no physical destruction could decrease ischemic apoptosis through decreasing membranous, mitochondrial and nuclear dysfunction.

### 2.4. Expression of Antiapoptotic Tie-2, Akt and Bcl-2

**Figure 6:**
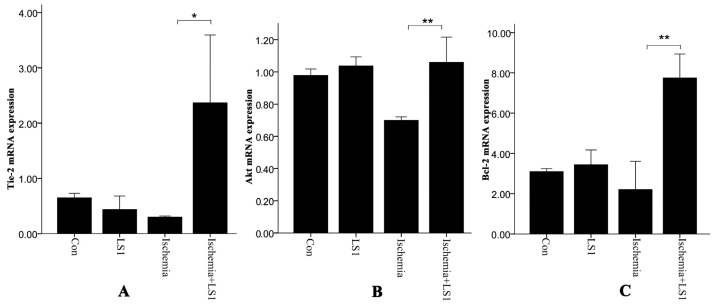
The statistics between groups about (**A**) *Tie-2*, (**B**) *Akt* and (**C**) *Bcl-2* gene expressions by real-time PCR.For each factor, there was no statistically significant difference between the Con group and LS1 group (*p* > 0.05), but there was a statistically significant difference between Ischemia group and Ischemia + LS1 group (* *p* < 0.05 or ** *p* < 0.01).

**Figure 7:**
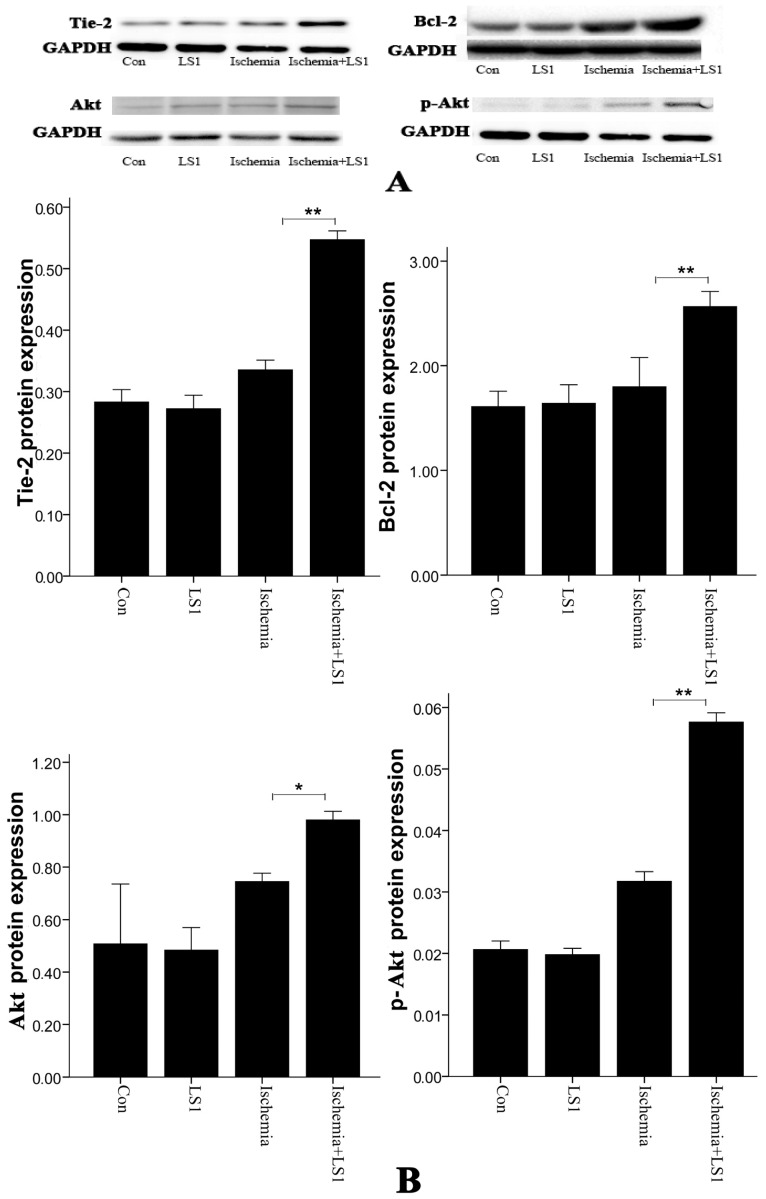
Effect of LS on Tie-2, Akt, p-Akt and Bcl-2 protein expressions by western blot and the apoptotic statistics between groups. (**A**) Tie-2, Akt, p-Akt and Bcl-2 protein expressions by western blot were normalized to GAPDH; (**B**) For Akt, p-Akt, Tie-2 and Bcl-2, there was not statistically significant difference between Con group and LS1 group (*p* > 0.05), but they statistically increased in Ischemia+LS1 group than Ischemia group. (* *p* < 0.05 or ** *p* < 0.01).

Real-time PCR and Western blot were used to test the mRNA and protein levels of Tie-2, Akt and Bcl-2 in Figures 6 and 7. With or without LS, rBMECs in normal culture condition had similar mRNA and protein expression (*p* > 0.05). Whereas in ischemic condition, additional stress upregulated genes of *Tie-2*, *Akt* and *Bcl-2* as well as proteins of Tie-2, Akt, Bcl-2 and phosphorylation-Akt (p-Akt) (* *p* < 0.05 or ** *p* < 0.01). Facing ischemic injury, LS stimulated Tie-2, Akt and Bcl-2 antiapoptotic activation.

### 2.5. Discussion

Laminar shear stress (LS) generated by cerebral blood flow (CBF) could attenuate rat brain microvascular endothelial cell (rBMECs) apoptosis under ischemic condition in our study. It was necessary to take measures to restore the damaged blood supply for ischemic penumbra after stroke [2–4].

Endothelial apoptosis is a major occurrence in ischemic penumbra,then it is significant to confer neuroprotection by targeting apoptotic alleviation in order to maintain the integrity of the blood-brain barrier and neurological function [14,15]. Apoptosis mainly occurs in normal physiologic process for metabolic turnover and homeostasis, while dysfunctional apoptosis in ischemic penumbra [12,15,26] would cause pathological brain damage. Fortunately, apoptosis is a programmed dynamic death process with certain gene and protein regulation so that interventions and treatments in experimental and clinic stroke models could alleviate or inhibit its occurrence or development. In our research, a simple and well-defined oxygen/glucose deprivation (OGD) system with glucose- and sodium pyruvate-free DMEM medium in mixture of 5% CO_2_/95% N_2_ (*v*/*v*) air *in vitro* was used to imitate ischemic injury [34]. We certified the existence of apoptotic rBMECs. However, cells had a strong self-regulation to confront apoptosis at first and the antiapoptotic molecules of Tie-2, Akt and Bcl-2 were ischemia-activated accordingly to show an expression peak in 2–4 h, so that there was no statistically apoptotic difference until ischemia 6 h (Figure 2). Of course, if ischemia kept on going, the regulating ability and molecular expression would gradually declined till disappeared with time (Figure 2). Therefore, taking aid measures with LS in 4–6 h was favorable.

Nowadays, *in vitro* simulation is still the primary means of hemodynamic study, as no appropriate manner to purely study the effect of LS *in vivo.* Parallel-plate flow chamber, a classic instrument widely used in the LS study [27–29], was adopted to give rBMECs stress. Stress magnitudes had a great influence on apoptosis. Many previous reports have shown a beneficial effect of high laminar shear stress in different diseases of atherosclerosis, pregnancy and genotoxicity than low shear stress [9,13,17,18,35]. Although there were no reports about the LS magnitudes of CBF for technological limitation, we might be sure that it was extremely lower than the venous system (1 to 6 dynes/cm^2^) and arterial system (10 to 70 dynes/cm^2^) [9]. Therefore, the applied 1 ± 0.05 dynes/cm^2^ should be mildly higher than normal CBF. We detected LS effects on apoptosis with three different methods of JC-1, PE Annexin V/7-AAD and Hoechst33258 staining. Differences were not obvious between the Con group and LS1 group, proving that the stress intensity almost had no additional mechanical injury to cells. When stress was added to ischemia 4–6 h, cells apoptotic amount was decreased to verify the mechanical mechanism for cerebral protection.

Conversion of mechanical signals into biochemical responses is essentially the unique mechanism of cellular mechanotransduction [10–12], so is the LS effect. It had been recognized that stress generally activated the critical mechanosensitive molecules on cells membranes at first; then the active molecules started a series of intracellular gene and protein reactions; at last, the organelles of cytoskeleton, cytoplasm, mitochondria, nuclei and so on, would be affected and changed [10–12]. Manners of JC-1, Hoechst33258 and PE Annexin V/7-AAD staining [20–22] not only calculated the rate of apoptosis, but also represented the program of mitochondrial depolarization, chromatin condensation and membrane permeability, respectively [12,19]. Mitochondrial depolarization and membrane permeability were the earlier events in apoptosis [19], while nuclear change was the apoptotic later features [26]. The changes of each sample were unequal with different manners for detecting different apoptotic stages, but the apoptotic trends between groups of three manners were parallel in our studies. Concerning the molecular, Tie-2, Bcl-2 and Akt, which were respectively maintain membranous, mitochondrial and nuclear norm were related. Their expression levels were unaffected in normal conditions, while dramatically upregulated in ischemic condition by LS.

Overall, antiapoptotic effects were not sensitive to CBF and LS in physiological conditions; in the case of ischemia-induced brain microvascular endothelial apoptosis happened, moderate LS could confront mitochondrial, membranous and nuclear dysfunction to ultimately alleviate apoptosis. LS was a credible source of endothelial protecting signals, and it was important to restore blood flow in ischemic stroke with appropriate treatments. Of course, the relationship between Tie-2, Bcl-2 and Akt was also unclear. Autophosphorylation of Tie-2 has been proven to have a strong antiapoptotic function through activating the downstream PI3K/Akt pathway [23,36–38]. Akt in the cytoplasm would also crosstalk with Bcl-2 bypass to achieve antiapoptotic signal amplification [39,40]. All of these previous studies implied the possibility for further investigation.

## 3. Experimental Section

### 3.1. Reagents

Dulbecco’s modified eagle medium (DMEM), fetal bovine serum (FBS), phosphate balanced solution (PBS) and bovine serum albumin (BSA) were purchased from Invitrogen (USA). Endothelial cell growth supplement (ECGS), heparin, l-glutamine, Hepes, insulin, Triton X-100, paraformaldehyde, H_2_O_2_, penicillin-streptomycin and laminin were from Sigma-Aldrich (USA). Collagenase/dispase was from Roche (USA).

### 3.2. Isolation and Culture of rBMECs

Based on previous methods [30,31], cerebral gray matter from male Sprague–Dawley (SD) rats (50–60 g) was chopped, homogenized and passed through 200 μm and 77 μm mesh. The tissue left on the 77 μm mesh screen was digested by means of 0.1% collagenase/dispase solution for 20 min at 37 °C, followed by resuspension in 25% BSA and centrifugation. The precipitation acquired was incubated in complete culture medium (high glucose DMEM, supplemented with 20% FBS, 100 μg/mL heparin, 100 μg/mL ECGS, 3.75 mg/mL Hepes, 0.2 U/mL insulin, 0.3 mg/mL l-glutamine, 100 U/mL penicillin and 100 μg/mL streptomycin, pH 7.2–7.4). We employed rBMECs at the third passage, which gained 95% purity in the present study.

### 3.3. Immunocytochemistry

Cells were fixed with 4% paraformaldehyde, incubated with von Willebrand factor (VWF, also called Factor VIII, BD Biosciences, Franklin Lakes, NJ, USA) [30] for one night at 4 °C, with FITC-conjugated secondary antibody (Jackson ImmunoResearch, West Baltimore Pike West Grove, PA, USA) for 60 min and DAPI (Vector Labs, Burlingame, CA, USA) for 15 min at room temperature. Treated cells were viewed by fluorescence microscope.

### 3.4. *In vitro* Angiogenesis Assay

Matrigel (BD Biosciences, Franklin Lakes, NJ, USA) placed evenly over 24-well culture plates was gelled at 37 °C for 30 min. rBMECs in 2% FBS-containing culture medium were seeded onto it and incubated at 37 °C for 18 h to form capillary-like structures [32] that we photographed under a phase contrast microscope.

### 3.5. Cell Treatments with Ischemia and LS

LS of 1 ± 0.05 dynes/cm^2^ was given to cells by a parallel-plate flow chamber (University of Shanghai for Science and Technology, China) [27–29]. rBMECs grown to confluence on glass coverslips were placed at the bottom of a notch in the chamber perfused with saturated 5% CO_2_/95% O_2_ (*v*/*v*) air and DMEM complete culture medium could flow through over the top of coverslips surface in a single direction. The machine could adjust the flow velocity and LS automatically. Simultaneously, cells were in ischemic condition with oxygen/glucose deprivation (OGD) of glucose- and sodium pyruvate-free DMEM (supplemented with no FBS) flow and 5% CO_2_/95% N_2_ (*v*/*v*) air.

### 3.6. WST-8 Assay

The viability of OGD rBMECs was analyzed by WST-8 assay with a Cell Counting Kit-8 (CCK-8) (Dojindo Laboratories, Kamimashiki-gun, Kumamoto, Japan) [33]. Ten microlitres CCK-8 solution was added per one milliliter of culture medium, incubated for 4 h at 37 °C and quantified by optical density at 450 nm using a spectrophotometer.

### 3.7. Hoechst 33258 Apoptotic Staining

Apoptosis was evaluated by Hoechst33258 (Beyotime, Jiangsu, China) staining [21]. Treated cells were stained with Hoechst 33258 (5 μg/mL) for 30 min at 4 °C in the dark and then imaged under a fluorescent microscope. The mean apoptotic ratio was assessed by three random fields in each well by image-pro plus software.

### 3.8. Mitochondrial Membrane Potentials Assay

JC-1 probe was employed to measure mitochondrial depolarization [20]. Cells were suspended in an equal volume of JC-1 staining solution (5 μg/mL) (Sigma, Gaithersburg, MD, USA) at 37 °C for 20 min. 10,000 cells at least in each sample were collected and analyzed for the emitted JC-1 fluorescence of red or green on flow cytometry.

### 3.9. PE Annexin V/7-AAD Apoptotic Analysis

Cells were suspended in binding buffer containing PE Annexin V and 7-AAD (BD Biosciences, Franklin Lakes, NJ, USA) at room temperature for 15 min in the dark. A minimum of 10,000 stained cells were immediately assayed on a flow cytometer. Data was analyzed with flowjo analysis software [22].

### 3.10. Real-Time PCR

Real-time PCR was adopted to analyze mRNA expression. Total RNA was extracted from cell cultures with TRIZOL reagent (Invitrogen, Faraday Avenue Carlsbad, CA, USA) and was followed by reverse transcription in accordance with the PrimeScript™ RT reagent Kit (TaKaRa, Otsu, Shiga, Japan). Real-time PCR was performed with a SYBR Green PCR Amplification Kit (TaKaRa, Otsu, Shiga, Japan). The data obtained were used to quantify the relative gene expression, with all samples normalized to β-actin. The primers used were as follows: Tie-2: forward, 5′-TCACAACAGCGTC TATCG-3′; reverse, 5′-ACCACCTCTCAACTTCCA-3′; Akt: forward, 5′-GCTCTTCTTCCACCTG TC-3′; reverse, 5′-GCCATAGTCGTTGTCCTC-3′; Bcl-2: forward, 5′-CCTGGCATCTTCTCCTTC-3′; reverse, 5′-GCTGACTGGACATCTCTG-3′; β-actin: forward, 5′-CCATTGAACACGGCATTG-3′; reverse, 5′-TACGACCAGAGGCATACA-3′.

### 3.11. Western Blot

Western blot was adopted to analyze protein expression. Protein was extracted from cells using RIPA lysis buffer (Beyotime, Jiangsu, China), separated using SDS-PAGE and transferred to PVDF membranes. Membranes were blocked with 3% BSA, incubated with primary antibodies (Akt, p-Akt and Bcl-2 from Cell Signaling Technology, USA; Tie-2 from Santa Cruz, CA, USA), incubated with peroxidase-conjugated secondary antibodies (Jackson ImmunoResearch, West Baltimore Pike West Grove, PA, USA) and, finally, visualized.

### 3.12. Statistical Analysis

Data was evaluated with a SPSS 20.0 statistical package. Values for each group were summarized as mean ± SD. One-way analysis of variance (ANOVA) was used to determine distinguished differences among groups. *p*-Values < 0.05 were considered statistically significant.

## 4. Conclusions

In this study, we found that appropriate LS generated by flow could attenuate rBMECs apoptosis under ischemic condition through decreasing membranous, mitochondrial and nuclear dysfunction. It proved that any therapy that could straightly restore blood flow and promote LS should be recommended as an excellent therapeutic strategy for ischemic stroke.
